# The impact of COVID-19 on adjusted mortality risk in care homes for older adults in Wales, UK: a retrospective population-based cohort study for mortality in 2016–2020

**DOI:** 10.1093/ageing/afaa207

**Published:** 2020-09-19

**Authors:** Joe Hollinghurst, Jane Lyons, Richard Fry, Ashley Akbari, Mike Gravenor, Alan Watkins, Fiona Verity, Ronan A Lyons

**Affiliations:** Health Data Research UK, Swansea University Medical School, Swansea, United Kingdom; Health Data Research UK, Swansea University Medical School, Swansea, United Kingdom; Administrative Data Research UK, Swansea University Medical School, Swansea, United Kingdom; Health Data Research UK, Swansea University Medical School, Swansea, United Kingdom; Administrative Data Research UK, Swansea University Medical School, Swansea, United Kingdom; National Centre for Population Health and Wellbeing Research, Swansea University Medical School, Swansea, United Kingdom; Health Data Research UK, Swansea University Medical School, Swansea, United Kingdom; Administrative Data Research UK, Swansea University Medical School, Swansea, United Kingdom; College of Medicine, Swansea University Medical School, Swansea University, UK, Singleton Park, Swansea, Wales SA2 8PP; Health Data Research UK, Swansea University Medical School, Swansea, United Kingdom; College of Medicine, Swansea University Medical School, Swansea University, UK, Singleton Park, Swansea, Wales SA2 8PP; Human and Health Sciences College, Swansea University, United Kingdom; Health Data Research UK, Swansea University Medical School, Swansea, United Kingdom; Administrative Data Research UK, Swansea University Medical School, Swansea, United Kingdom; National Centre for Population Health and Wellbeing Research, Swansea University Medical School, Swansea, United Kingdom

**Keywords:** COVID-19, care homes, mortality, frailty, older people

## Abstract

**Background:**

mortality in care homes has had a prominent focus during the COVID-19 outbreak. Care homes are particularly vulnerable to the spread of infectious diseases, which may lead to increased mortality risk. Multiple and interconnected challenges face the care home sector in the prevention and management of outbreaks of COVID-19, including adequate supply of personal protective equipment, staff shortages and insufficient or lack of timely COVID-19 testing.

**Aim:**

to analyse the mortality of older care home residents in Wales during COVID-19 lockdown and compare this across the population of Wales and the previous 4 years.

**Study Design and Setting:**

we used anonymised electronic health records and administrative data from the secure anonymised information linkage databank to create a cross-sectional cohort study. We anonymously linked data for Welsh residents to mortality data up to the 14th June 2020.

**Methods:**

we calculated survival curves and adjusted Cox proportional hazards models to estimate hazard ratios (HRs) for the risk of mortality. We adjusted HRs for age, gender, social economic status and prior health conditions.

**Results:**

survival curves show an increased proportion of deaths between 23rd March and 14th June 2020 in care homes for older people, with an adjusted HR of 1.72 (1.55, 1.90) compared with 2016. Compared with the general population in 2016–2019, adjusted care home mortality HRs for older adults rose from 2.15 (2.11, 2.20) in 2016–2019 to 2.94 (2.81, 3.08) in 2020.

**Conclusions:**

the survival curves and increased HRs show a significantly increased risk of death in the 2020 study periods.

## Key points

- Routinely collected data can be used for rapid up-to-date analysis of mortality.
- Older people in care homes are vulnerable to the outbreaks of infectious disease.
- Increased mortality risk in care homes during the COVID-19 pandemic (2020) compared with previous years (2016–2019).

## Introduction

### Background

Mortality in care homes has had a prominent worldwide focus during the COVID-19 outbreak [1,2] but few detailed analyses have been conducted. Care homes are a keystone of adult social care. They provide accommodation and care for those needing substantial help with personal care, but more than that, they are people’s homes [2,3]. In 2016, there were 11,300 care homes in the UK, with a total of 410,000 residents [4].

Care home markets vary across the 22 local government authorities in Wales in the supply, ownership and size of care homes [5]. While the main providers are single operators of one home, care homes are also owned by local authorities, small operators (2–3 care homes) and large operators (4 or more care homes) [5]. There is a small number of not for profit providers. Following a wide reaching inquiry into quality of life and care in care homes, the Older Person’s Commissioner for Wales concluded that ‘too many older people living in care homes have an unacceptable quality of life’ [6]. The Commissioner’s expectations for change were far ranging and included greater investment in the care home sector, staff development, recalibrating a human rights focus, quality reporting and provision of a range of health services [6].

Within care homes people live in proximity and may live with frailty and many different health conditions, making them susceptible to outbreaks of infectious disease [3]. COVID-19 is described by Lithande et al. as ‘… a dynamic, specific and real threat to the health and well-being of older people’ (2020, p.10) [7]. The impacts of COVID-19 on this sub-population have been reported widely in both international and UK media and in a growing peer-reviewed literature.

Multiple and interconnected challenges face the care home sector in the prevention and management of outbreaks of COVID-19 [2]. In the literature, these challenges are reported to include staff shortages [1,2], insufficient or lack of timely COVID-19 testing [2,8] and poor access to personal protective equipment [1,2,8,9]. Related clinical challenges include older adults with COVID-19 being asymptomatic, or not displaying expected symptoms [1,2,7,8]. Once there is an outbreak, the disease can spread quickly within a care home setting and be difficult to contain [8–10]. A further challenge is in managing the impact of practices to shield care home residents and isolate those who are infected. These practices can result in social isolation from families, friends and communities, with negative impacts on health and wellbeing [2,7]. Set against these challenges is the caring, innovative and resilient response of care home staff and residents in managing the situations they face [11].

This confluence of events in the context of the pandemic, and impacts for residents, their families and care home staff, has been framed as a human rights issue [12]. In the UK, it is argued that underinvestment in the care home sector and a poor interface with the health sector led to ill-informed policies, for example the rapid hospital discharge policies in the early period of the lockdown [9,13–15]. COVID-19 is a rapidly evolving complex issue requiring near real-time data, analyses and a multidisciplinary team to devise, implement and evaluate a wide variety of inter- and cross-sectorial interventions to minimise population harm.

The use of existing anonymised routinely collected longitudinal data can help to provide rapid access to large-scale data for studies and provide robust evidence for commissioning decisions and policy [16]. In this study, we utilise the Secure Anonymised Information Linkage (SAIL) Databank [17–19] to investigate mortality in care homes in Wales in the initial phase of the UK lockdown and compare this with corresponding data from the four most recent years to estimate excess mortality.

### Research questions

We aimed to compare the mortality risk for older care home residents (60+) in Wales for each year between 2016 and 2020. To do this, we performed the following two sets of analyses.

- How does mortality in care homes for older adults compare between 2016 and 2020?
- How does care home mortality for older adults compare between 2020 and 2016–2019 in the context of the population of Wales?

## Methods

### Study design

We used anonymised Electronic Health Records and administrative data from the SAIL Databank to create a cross-sectional cohort study.

### Data sources

Our cohorts were created using data held within the SAIL Databank [17–19]. The SAIL Databank contains longitudinal anonymised administrative and healthcare records for the population of Wales. The anonymisation is performed by a trusted third party, the National Health Service (NHS) Wales Informatics Service (NWIS). The SAIL Databank has a unique individual anonymised person identifier known as an Anonymous Linking Field (ALF) and unique address anonymised identifier known as a Residential ALF (RALF) [20] that are used to link between data sources at individual and residential levels, respectively. Individual linking fields, nested within residential codes, are contained in the anonymised version of the Welsh Demographic Service Dataset (WDSD), replacing the identifiable names and addresses of people registered with a free-to-use General Practitioner service.

Our cohort of older care home residents was determined by linking to an existing index for anonymised care home addresses from a previous project [21] and utilising the WDSD for address changes. We determined if someone was a care home resident by linking their de-identified address information to the residences indexed as a care home in the WDSD. The anonymised care home index was created using the Care Inspectorate Wales (CIW) [22] data source from 2018 and assigning a Unique Property Reference Number (UPRN) to each address [23]. We included care homes with a classification of either care homes for older adults or care homes for older adults with nursing in our list. The UPRN was double-encrypted into a project level RALF and uploaded into SAIL to create a deterministic match to the WDSD. From an analysis perspective, both residents and care homes are de-identified prior to any analysis.

### Setting and participants

To answer our research questions, we created separate data sets, both with different settings and participants.

- Initially, we focussed on the phase of the UK lockdown for the COVID-19 pandemic, from 23rd March to 14th June 2020, and compared the mortality risk of care home residents to those in the same period (23rd March—14th June) in each of the previous years from 2016 to 2019. Individuals in Wales aged 60+ years identified in the SAIL Databank as a resident in a care home on 23rd March in one of our study years (2016–2020). We created 5 cohorts, one for each year of study, and treated these as independent.
- We compared the mortality risk of being in a care home at the population level between 1 January 2020—30 April 2020 and 1 January 2016—31 December 2019. We used the WDSD to create population wide cohorts; this included all individuals resident in Wales from 2016 to 2020. We stratified the dataset in to four sub-groups for comparison as follows.
  (i) Non-care home residents, resident in Wales on 1/1/2016. Residents were followed up until they moved out of Wales, died, or 31/12/2019.
  (ii) Non-care home residents, resident in Wales from 1/1/2020 to 30/4/2020. Residents were followed up until they moved out of Wales, died, or 30/04/2020.
  (iii) Care home residents, resident in a care home for older people in Wales on 1/1/2016. Residents were followed up until they moved out of Wales, died, or 31/12/2019.
  (iv) Care home residents, resident in a care home for older people in Wales from 1/1/2020 to 30/4/2020. Residents were followed up until they moved out of Wales, died, or 30/04/2020.

### Hospital Frailty Risk Score

The Hospital Frailty Risk Score (HFRS) was developed using Hospital Episode Statistics (HES), a database containing details of all admissions, Emergency Department attendances and outpatient appointments at NHS hospitals in England, and validated on over one million older people using hospitals in 2014/15 [24]. The HFRS uses the International Classification of Disease version 10 [25] (ICD-10) codes to search for specific conditions from secondary care. A weight is then applied to the conditions and a cumulative sum is used to determine a frailty status of: low, intermediate or high. We additionally included an HFRS score of ‘No score’ for people who had not been admitted to hospital in the look back period. We calculated the HFRS using the Patient Episode Database for Wales, the Welsh counterpart to HES, on the entry date for each of our studies, with a 2-year look back of all hospital admissions recorded in Wales.

### Outcome of interest—mortality

We used a combination of the Office of National Statistics (ONS) Annual District Death Extract (ADDE), WDSD and Consolidated Death Data Source (CDDS) to link historic and current mortality information. The CDDS is a combination of the ONS mortality data along with the death records found in the Master Patient Index, and was used to identify deaths in 2020; the ADDE and WDSD were used to identify deaths from 2016 to 2019.

### Demographics

Additional demographic information was taken from the WDSD. The WDSD includes the week of birth, the 2011 Lower layer Super Output Area used to assign the 2019 Welsh Index of Multiple Deprivation (WIMD), and gender. We calculated the age of individuals on the study start date for each of our analyses.

### Statistical methods

#### Kaplan–Meier survival curves

For our first analysis, the Kaplan–Meier survival function was estimated from 23rd March to 14th June for each year of care home residency (2016–2020).

#### Cox regression

Cox regression was used to determine hazard ratios (HRs) for mortality with 95% confidence intervals. Adjusted HRs included: the cohort year, care home residency, age, gender, HFRS and WIMD (2019 version). We included covariates using a stepwise approach, enabling us to see the impact of the additional variables on the overall model. We included a cluster level effect for each residence. Computation restrictions meant that we were unable to include a cluster level effect for the second analysis.

## Results

### Research question 1: how does mortality in care homes for older adults compare between 2016 and 2020?

**Figure 1:**
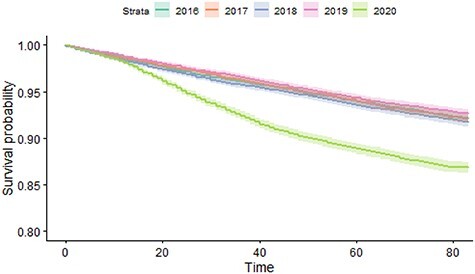
The figure shows the Kaplan–Meier Curves for each cohort (2016–2020). The horizontal axis refers to the time in number of days after the 23rd March up until the 14th June for each year.

We analysed over 12,000 individuals per year in more than 500 care homes for older adults. Demographic information remained consistent across years, but we observed a much higher proportion of deaths in 2020 when compared with previous years. We present the descriptive data for the cohorts in Table 1, Kaplan–Meier survival curve in Figure 1 and Cox proportional hazards models in Table 2.

**Table 1:** Demographic information for each of the care home cohorts stratified by year

Cohort year	2016	2017	2018	2019	2020
Individuals (*N*)	13,950	13,481	12,707	12,642	12,568
Care homes	572	572	572	554	544
Deaths (n)	1,103	1,076	1,059	941	1,655
Deaths (%)	7.91%	7.98%	8.33%	7.44%	13.17%
Age					
Mean age (sd)	85.3 (8.4)	85.3 (8.3)	85.2 (8.4)	85.2 (8.4)	85.2 (8.5)
Gender					
Female	72.0%	71.5%	71.4%	71.5%	70.8%
HFRS					
No score	40.0%	39.8%	40.2%	39.9%	40.8%
Low	10.3%	9.7%	9.2%	8.9%	9.0%
Intermediate	25.2%	24.6%	24.0%	23.6%	23.3%
High	24.4%	25.8%	26.6%	27.7%	26.9%
WIMD 2019					
1. Most deprived	15.5%	15.4%	15.3%	15.7%	15.2%
2.	22.0%	22.0%	21.3%	21.8%	21.8%
3.	22.1%	22.3%	21.8%	21.4%	21.1%
4.	20.1%	20.6%	20.8%	20.2%	20.8%
5. Least deprived	20.3%	19.7%	20.8%	21.0%	21.1%

**Table 2:** The table shows the HRs for adjusted Cox proportional hazards models with a cluster effect for each care home

Models (aggregate inclusion)	Cohort year	Age	Gender	HFRS	WIMD
Cohort year (baseline: 2016)					
2017	1.01 (0.93, 1.09)	1.01 (0.93, 1.10)	1.01 (0.93, 1.09)	1.00 (0.93, 1.09)	1.00 (0.93, 1.09)
2018	1.06 (0.97, 1.15)	1.06 (0.97, 1.16)	1.06 (0.97, 1.16)	1.05 (0.97, 1.15)	1.05 (0.97, 1.15)
2019	0.94 (0.86, 1.03)	0.94 (0.86, 1.03)	0.94 (0.86, 1.03)	0.93 (0.85, 1.01)	0.93 (0.85, 1.01)
2020	1.72 (1.56, 1.91)	1.73 (1.56, 1.92)	1.73 (1.56, 1.91)	1.72 (1.55, 1.90)	1.72 (1.55, 1.90)
Age (continuous 60+)					
Age	–	1.03 (1.03, 1.04)	1.04 (1.03, 1.04)	1.04 (1.03, 1.04)	1.04 (1.03, 1.04)
Gender (baseline: female)					
Male	–	–	1.44 (1.35, 1.53)	1.39 (1.31, 1.47)	1.39 (1.31, 1.47)
HFRS (baseline: no score)					
Low	–	–	–	1.08 (0.96, 1.20)	1.07 (0.96, 1.20)
Intermediate	–	–	–	1.30 (1.21, 1.39)	1.30 (1.21, 1.39)
High	–	–	–	1.65 (1.54, 1.76)	1.64 (1.54, 1.75)
WIMD 2019 (baseline: 1. Most deprived)					
	–	–	–	–	0.98 (0.95, 1.00)
	–	–	–	–	–
Concordance	0.555 (s.e. 0.005)	0.595 (s.e. 0.005)	0.606 (s.e. 0.004)	0.621 (s.e. 0.004)	0.622 (s.e. 0.004)

#### Sensitivity analyses

To check the influence of individuals being present in more than one cohort, we have included the number of individuals who are common across each study year in Appendix 1 in the Supplementary data. We independently calculated adjusted HRs for the 2020 cohort against each of the study years; the results are presented in Appendix 2 in Supplementary data. We also present the HRs without a cluster level effect in Appendix 3 in Supplementary data.

### Research question 2: how does care home mortality for older adults compare between 2020 and 2016–2019 in the context of the population of Wales?

Our extended analysis included over three million individuals in the 2016–2019 and 2020 periods of study. The demographic information of each of the cohorts is presented in Table 3 and the corresponding regression model results are displayed in Table 4. Additional models with the individual covariates are presented in Appendix 4 in Supplementary data.

**Table 3:** Population wide cohort demographics for care home and non-care home residents in 2020 and 2016–2019

Cohort	1.Non-care home residents: 2016–2019	2.Non-care home residents: 2020	3.Care home residents: 2016–2019	4.Care home residents: 2020
Individuals (*N*)	3,072,352	3,259,087	14,680	13,417
Residences (*N*)	1,190,331	1,195,568	591	565
Cohort start date	01/01/2016	01/01/2020	01/01/2016	01/01/2020
Cohort end date	31/12/2019	30/04/2020	31/12/2019	30/04/2020
Maximum period (days inclusive)	1,461	121	1,461	121
Deaths in period	4.2%	0.4%	77.9%	17.6%
(Deaths %/days)	0.00284%	0.00326%	0.05333%	0.14,586%
Mean age (sd)	41.1 (23.5)	41.6 (23.7)	84.2 (11.0)	84.1 (10.9)
Gender				
Female	50.0%	50.0%	71.2%	69.9%
HFRS				
No score	89.6%	88.4%	41.0%	41.1%
Low	8.3%	9.2%	10.2%	8.7%
Intermediate	1.7%	2.0%	24.7%	23.7%
High	0.4%	0.5%	24.1%	26.5%
WIMD 2019 quintile				
1. Most deprived	20.4%	19.1%	15.5%	14.9%
2	19.9%	18.5%	22.1%	21.3%
3	20.1%	18.4%	22.1%	20.6%
4	19.7%	18.1%	20.0%	20.4%
5. Least deprived	19.9%	18.3%	20.3%	20.3%
Missing WIMD	0.0%	7.7%	0.0%	2.5%

**Table 4:** Cox regression results for the extended observation period. Adjustments for age, gender, WIMD and the HFRS are included. The baseline cohort was non-care home residents in the period 2016–2019

	Cohort model	Adjusted model
Baseline—1. non-care home residents: 2016–2019		
2.Non-care home residents: 2020	1.11 (1.09, 1.14)	0.99 (0.96, 1.01)
3.Care home residents: 2016–2019	36.85 (36.15, 37.57)	2.15 (2.11, 2.20)
4.Care home residents: 2020	53.79 (51.48, 56.21)	2.94 (2.81, 3.08)
Age	–	1.09 (1.09, 1.09)
Gender (baseline Female)		
Male	–	1.43 (1.41, 1.44)
WIMD 2019 (baseline: 1. Most deprived)	–	0.91 (0.90, 0.91)
HFRS (Baseline—No score)	–	
Low	–	1.95 (1.92, 1.98)
Intermediate	–	3.58 (3.52, 3.63)
High	–	4.90 (4.80, 5.01)
Concordance	0.554 (s.e. = 0.001)	0.915 (s.e. < 0.001)

## Discussion

When compared with previous years and after adjustment for age, sex, deprivation and HFRS, our results show substantial excess mortality in care home residents during the first phase of the COVID-19 lockdown. The baseline demographics shown in Table 1 show a consistent trend across each study year with the exception of mortality in 2020. This is consistent with the diverging Kaplan–Meier curves displayed in Figure 1 and the increased HRs for the cohort year presented in Table 2. The HR for the cohort year remains statistically insignificant for 2017/18/19 when compared with 2016, but the HR for 2020 is consistently greater than 1.7 for each of the models presented. We adjusted for age, gender, HFRS and WIMD in the models. It was found that age, gender (male) and increasing HFRS led to an increased HR for mortality. This is consistent with previous studies, where frailty has been shown to be associated with increased mortality [26–28]. We found that the WIMD was a significant factor in the population level analyses. The cluster effect term indicated that there was a variation between care homes; this is likely because of differences in the case mix of care home residents and the varying exposure to COVID cases.

The KM curves may indicate a flattening of the divergence in mortality in more recent weeks. We plan to repeat these analyses as more data become available. Inclusion of data on the timing of interventions and policy changes, both across the health care system and in care homes, would help understand the effectiveness of different approaches on reducing transmission of infection and clinical outcomes.

Table 3 details the differences in demographic information between care home residents and the general population. Specifically, the care home residents are more likely to be women and have increased mortality, frailty and age. The analysis in Table 4 indicated a higher risk of mortality for care home residents compared with the general population. The analysis also showed an increased risk of mortality in care homes in 2020 compared with the 2016–2019 counterparts. Interestingly, the mortality risk of the general population in 2020 compared with 2016–2019 was statistically insignificant, further highlighting the increased risk of mortality in care homes in 2020.

We have demonstrated that using anonymised data linkage; we can repurpose existing cohorts and methodologies to directly inform social care policy. The results from this analysis have been used to describe the impact of COVID-19 on mortality in care homes in Wales, while informing national efforts to prepare for potential second waves in winter 2020–2021.

## Limitations

Although we used a consistent list of anonymised care home addresses, there is a varying number of care homes included in each year of study. This is due to the list of care homes being created from the 2018 extract from CIW, and care homes being opened and closed. We aimed to mitigate bias in our comparisons by using a consistent list across study years.

Our cohorts were created at cross-sectional time points; this means that individuals may appear in more than one cohort. Although we calculated the covariates at the individual level at the start of each cohort interval, there may still remain correlation between the cohorts. The WIMD assigned to individuals is based on their current residence; in the case of care home residents, this may not reflect the historic deprivation accrued across the life course as it may in more community-based individuals.

## Conclusions

We performed a retrospective population-based cohort study, comparing the mortality risk in care homes between 2016 and 2020. It was found that the mortality risk in care homes has increased significantly in 2020 compared with previous years. The conclusion of increased mortality risk in 2020 remained the same when we included additional demographic variables, the HFRS, and increased the observation window.

## Supplementary Material

aa-20-0917-File002_afaa207
