# Supplementary material for: The impact of COVID-19 on adjusted mortality risk in care homes for older adults in Wales, UK: a retrospective population-based cohort study for mortality in 2016–2020
Source: Age Ageing. 2020 Sep 19;50(1):25–31. doi: 10.1093/ageing/afaa207 (PMC7546151; doi:10.1093/ageing/afaa207)
Supplement: aa-20-0917-File002_afaa207 [file aa-20-0917-file002_afaa207.docx]

The Impact of COVID-19 on Adjusted Mortality Risk in Care Homes for Older Adults in Wales, United Kingdom: A retrospective population-based cohort study for mortality in 2016-2020

**SUPPLEMENTARY DATA**

- Appendix 1. Number of individuals common across cohort years
- Appendix 2. Cox regression results comparing care home mortality in 2020 to each of the previous year’s independently
- Appendix 3. Cox regression results for mortality in care homes without a cluster effect for each care home included
- Appendix 4. Full model derivation for the population wide cohorts comparing care home and non-care home residents in 2016-2019 and 2020.

Appendix 1. Number of individuals common across cohort years

| **Year** | **2016** | **2017** | **2018** | **2019** | **2020** |
| --- | --- | --- | --- | --- | --- |
| **2016** | 13950 | - | - | - | - |
| **2017** | 9097 | 13481 | - | - | - |
| **2018** | 5989 | 8670 | 12707 | - | - |
| **2019** | 4062 | 5843 | 8455 | 12642 | - |
| **2020** | 2812 | 4021 | 5809 | 8493 | 12568 |

Appendix 2. Cox regression results comparing care home mortality in 2020 to each of the previous year’s independently

| **Comparator** | **2016** | **2017** | **2018** | **2019** |
| --- | --- | --- | --- | --- |
| Age | 1·034 (1·029,1·039) | 1·037 (1·032,1·042) | 1·037 (1·031,1·042) | 1·033 (1·028,1·038) |
| 2020 | 1·712 (1·546,1·896) | 1·708 (1·553,1·879) | 1·626 (1·47,1·799) | 1·847 (1·667,2·046) |
| Male | 1·47 (1·355,1·594) | 1·476 (1·358,1·604) | 1·435 (1·325,1·553) | 1·369 (1·256,1·493) |
| Low | 1·048 (0·89,1·234) | 1·129 (0·971,1·313) | 1·052 (0·889,1·246) | 1·163 (0·98,1·379) |
| Intermediate | 1·244 (1·13,1·37) | 1·225 (1·106,1·357) | 1·218 (1·103,1·344) | 1·202 (1·084,1·332) |
| High | 1·602 (1·46,1·758) | 1·687 (1·535,1·853) | 1·563 (1·417,1·724) | 1·595 (1·447,1·758) |
| WIMD | 0·983 (0·944,1·024) | 0·976 (0·933,1·02) | 0·978 (0·938,1·019) | 0·987 (0·945,1·03) |
| - | - | - | - | - |
| Observations | 26518 | 26049 | 25275 | 25210 |
| Events | 2758 | 2731 | 2714 | 2596 |
| Removed | 0 | 0 | 0 | 0 |
| - | - | - | - | - |
| Concordance | 0·626 (s·e· 0·006 ) | 0·632 (s·e· 0·005 ) | 0·621 (s·e· 0·006 ) | 0·627 (s·e· 0·006 ) |

Appendix 3. Cox regression results for mortality in care homes without a cluster effect for each care home included

| Coefficients |  |  |  |  |  |
| --- | --- | --- | --- | --- | --- |
| 2017 | 1·009 (0·928,1·097) | 1·01 (0·929,1·099) | 1·009 (0·928,1·098) | 1·004 (0·923,1·092) | 1·004 (0·923,1·092) |
| 2018 | 1·058 (0·972,1·151) | 1·063 (0·977,1·157) | 1·062 (0·976,1·155) | 1·053 (0·968,1·146) | 1·054 (0·968,1·146) |
| 2019 | 0·938 (0·86,1·023) | 0·942 (0·864,1·028) | 0·94 (0·862,1·025) | 0·928 (0·851,1·012) | 0·928 (0·851,1·013) |
| 2020 | 1·722 (1·596,1·858) | 1·731 (1·604,1·868) | 1·726 (1·6,1·863) | 1·715 (1·589,1·85) | 1·716 (1·59,1·852) |
| Age | - | 1·031 (1·028,1·035) | 1·036 (1·033,1·04) | 1·036 (1·033,1·04) | 1·037 (1·033,1·04) |
| Male | - | - | 1·437 (1·358,1·52) | 1·388 (1·312,1·469) | 1·388 (1·311,1·468) |
| Low | - | - | - | 1·075 (0·971,1·189) | 1·074 (0·97,1·188) |
| Intermediate | - | - | - | 1·296 (1·211,1·388) | 1·295 (1·209,1·387) |
| High | - | - | - | 1·647 (1·546,1·754) | 1·643 (1·543,1·75) |
| WIMD | - | - | - | - | 0·976 (0·958,0·995) |
| - | - | - | - | - | - |
| Observations | 65348 | 65348 | 65348 | 65348 | 65348 |
| Events | 5834 | 5834 | 5834 | 5834 | 5834 |
| Removed | 0 | 0 | 0 | 0 | 0 |
| - | - | - | - | - | - |
| Concordance | 0·555 (s·e· 0·004 ) | 0·595 (s·e· 0·004 ) | 0·606 (s·e· 0·004 ) | 0·621 (s·e· 0·004 ) | 0·622 (s·e· 0·004 ) |

Appendix 4. Full model derivation for the population wide cohorts comparing care home and non-care home residents in 2016-2019 and 2020.

|  | Cohort | Age | Gender | WIMD | HFRS | Full |
| --- | --- | --- | --- | --- | --- | --- |
| Baseline - 0. non-carehome residents 2016 |  |  |  |  |  |  |
| 1.Non-care 2020 | 1·112 (1·085,1·14) | 1·061 (1·035,1·088) | 1·112 (1·085,1·14) | 1·066 (1·0393,1·0935) | 1·057 (1·031,1·083) | 0·9885 (0·9636,1·014) |
| 2.2016 care homes | 36·85 (36·147,37·57) | 3·441 (3·372,3·511) | 37·557 (36·836,38·293) | 36·9223 (36·2177,37·6405) | 5·711 (5·582,5·843) | 2·1529 (2·1075,2·1993) |
| 3.2020 care homes | 53·793 (51·48,56·21) | 4·763 (4·556,4·979) | 54·727 (52·371,57·19) | 54·2143 (51·8634,56·6718) | 7·78 (7·431,8·144) | 2·9403 (2·81,3·0766) |
| Age |  | 1·1 (1·099,1·1) |  |  |  | 1·0922 (1·0917,1·0926) |
| Gender (baseline Female) |  |  |  |  |  |  |
| Male |  |  | 1·087 (1·076,1·098) |  |  | 1·4254 (1·4109,1·44) |
| WIMD 2019 (baseline: 1. Most deprived) |  |  |  | 0·9801 (0·9766,0·9836) |  | 0·9054 (0·9021,0·9087) |
| Hospital Frailty Risk Score (Baseline - No score) |  |  |  |  |  |  |
| Low |  |  |  |  | 3·826 (3·777,3·877) | 1·9506 (1·9249,1·9765) |
| Intermediate |  |  |  |  | 14·459 (14·245,14·675) | 3·5779 (3·5239,3·6328) |
| High |  |  |  |  | 25·279 (24·732,25·838) | 4·9045 (4·8045,5·0065) |
|  |  |  |  |  |  |  |
| Concordance | 0·554 (se = 0·001) | 0·901 (se = 0) | 0·562 (se = 0·001) | 0·56 (se = 0·001) | 0·72 (se = 0·001) | 0·915 (se = 0) |
